# Giant Neurocysticercosis: A Rare Medical Condition

**DOI:** 10.7759/cureus.71090

**Published:** 2024-10-08

**Authors:** Jorge Zumaeta, Camilo Contreras, Paola Tapia, Diego Morales, Noe Santiago Rea, Jose Valerio

**Affiliations:** 1 Neurological Surgery, Latinoamerica Valerio Foundation, Weston, USA; 2 Vascular, Tumors and Functional Neurosurgery Service, Guillermo Almenara Irigoyen National Hospital, Lima, PER; 3 Neurosurgery, Arzobispo Loayza National Hospital, Lima, PER; 4 Neurological Surgery, Palmetto General Hospital, Hialeah, USA; 5 Neurological Surgery, Miami Neuroscience at Larkin Community Hospital, South Miami, USA

**Keywords:** central nervous system, craniotomy, intracranial hypertension, neurocysticercosis, parasite infection

## Abstract

Neurocysticercosis is the most common parasitic infestation of the central nervous system and is often asymptomatic. A giant presentation, defined as a cyst measuring 4 cm or more in its largest dimension, is a rare entity. The mass effect of such lesions can lead to neurological deterioration, making surgical resection necessary. We present three cases of neurocysticercosis with giant cystic lesions located intracranially, which caused significant mass effects. The patients primarily exhibited impaired consciousness, motor deficits, and seizures. All three patients underwent craniotomy for the resection of the cystic lesions, resulting in favorable outcomes and recovery of neurological function. The management of giant intracranial neurocysticercosis with substantial mass effect should be surgical. Adequate surgical resection can lead to significant neurological recovery for the patient.

## Introduction

Cerebral neurocysticercosis is a parasitic infection of the central nervous system (CNS) [1-3]. It is the most common cause of acquired epilepsy in low-income countries. Cysticercosis is endemic in areas such as Latin America, parts of Oceania, Eastern Europe, Asia, and Africa [2]. According to the World Health Organization, 50 million people worldwide have neurocysticercosis, and it leads to death in 50,000 cases each year [4]. The incubation period can be months to decades, with a median of 3.5 years; however, periods greater than 10 years have been reported [5]. Neurocysticercosis is characterized by the formation of intracranial cysts from the larva of *Taenia solium*, clinically manifested as epileptic seizures as its main symptom, and in rare occasions as progressive intracranial hypertension, which is acquired by ingesting the eggs of the parasite [6].

In the life cycle of *T. solium*, humans are usually infected by its adult form. The coexistence between pigs and humans, plus poor hygiene, causes humans to ingest parasite eggs accidentally; these pass through the intestinal wall and reach tissues such as the brain, where larval cysts develop. These cysts are mostly destroyed by the immune system [7,8]. These cysts, based on their location, can be classified as subarachnoid, parenchymal, ventricular, and spinal, according to the evolutionary phase and radiological findings [9]. Neurocysticercosis is divided into five stages: noncystic, vesicular, vesicular-colloidal, nodular-granulomatous, and nodular-calcified [10]. It is treated pharmacologically in its active phase with corticosteroids and anthelmintic drugs (albendazole or praziquantel) [11].

Although most cases of neurocysticercosis are managed with antiparasitic and anti-inflammatory drugs, surgical treatment is indicated in those cases that involve spinal, intraventricular, or subarachnoid cysts in association with hydrocephaly, giant cysts of more than 40 mm with motor deficit or persistence of intracranial hypertension despite medical treatment [1,12-14].

## Case presentation

**Figure 1:**
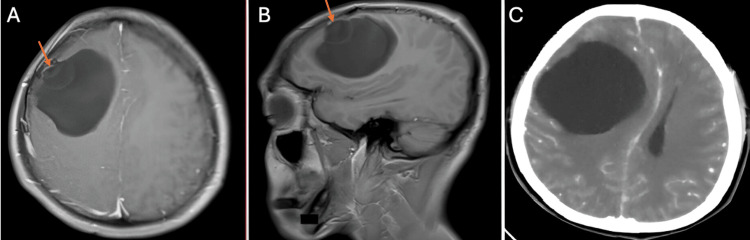
MRI showing a giant cysticercus cyst with scolex inside (orange arrow) (A,B). Head CT showing the cystic mass exerting a severe mass effect, generating deviation from the midline (C) MRI: magnetic resonance imaging; CT: computed tomography

**Figure 2:**
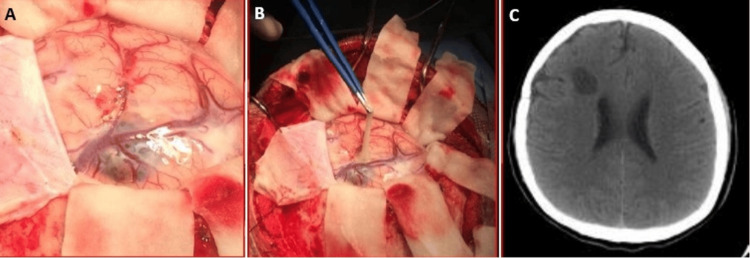
Intraoperative images showing chemical mass with scolex at the cortical level (A) and the removal of cysticercus cyst (B). CT head control showing the absence of cysticercus cyst after surgery (C) CT: computed tomography

The first case is a 28-year-old man from the southern coast of Peru. He had been ill for a month, characterized by a right frontal headache that progressed in intensity, accompanied by nausea and weakness on the left side of the body. On examination, he was a patient with a state of consciousness of 15 points on the Glasgow Coma Scale with moderate left hemiparesis. Imaging examinations showed a 7 cm × 7 cm right frontal cystic lesion, with mass effect, deviating the midline and collapsing the right lateral ventricle. It did not capture contrast, and no perilesional edema was evident. Brain magnetic resonance imaging (MRI) showed the presence of a scolex, which corresponds to the parasite head (Figures 1A-1C). The Western blot test for cysticercosis was performed, resulting in a positive result. The patient was scheduled for surgery, performing a right frontal craniotomy and removal of the cystic lesion (Figures 2A, 2B). He had a favorable postoperative evolution, recovering strength in his left-sided hemiparesis. He was discharged early, without the motor deficit, and indicated treatment with albendazole. The control tomography showed complete removal of the cyst and the absence of complications (Figure 2C).

**Figure 3:**
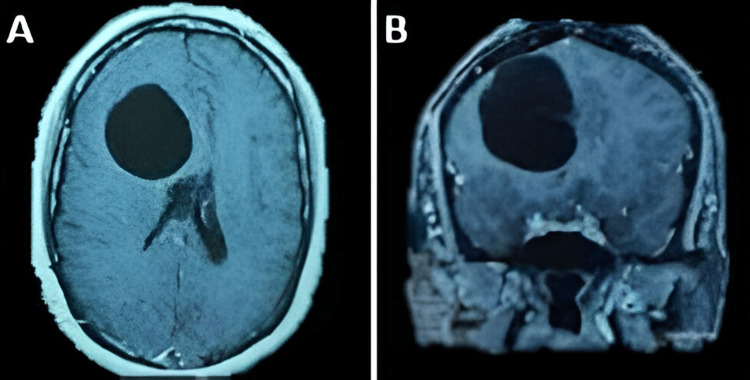
Right frontal lobulated giant neurocysticercosis. Brain MRI shows giant neurocysticercosis at the right frontal level, multipartitioned in the axial (A) and coronal (B) planes MRI: magnetic resonance imaging

**Figure 4:**
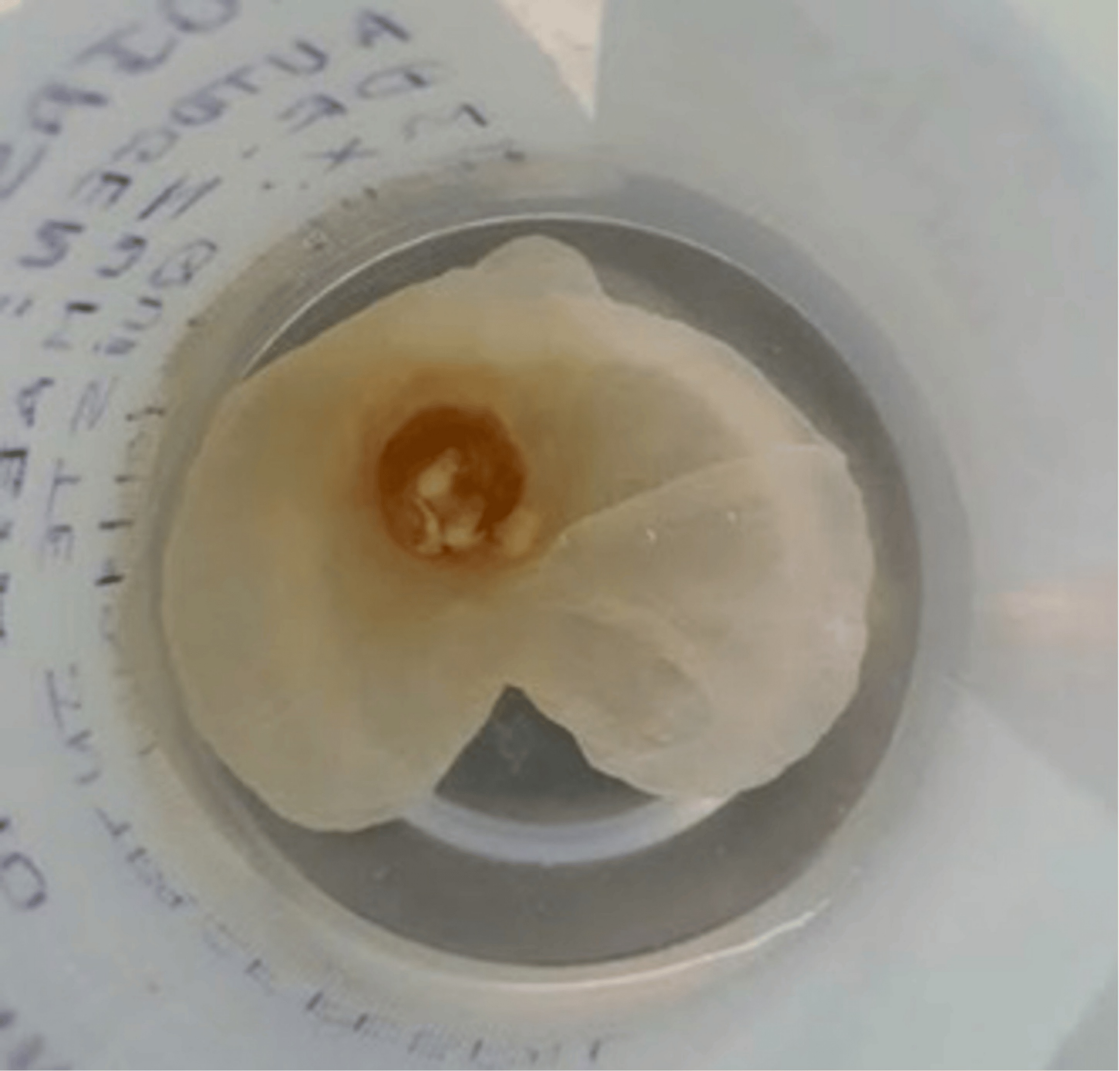
Macroscopic image of cysticercus after its removal

The second case is a 40-year-old female patient from the central coast of Peru. She was admitted to the hospital with a headache of progressive intensity associated with nausea and vomiting. On examination, the patient was drowsy and had left hemiparesis. Imaging studies showed a 6 cm × 5 cm multipartitioned right frontal cystic lesion (Figures 3A, 3B). The Western blot study for cysticercosis was positive. Given the persistence of impaired consciousness, an emergency procedure was scheduled for a right frontal craniotomy plus excision of the cystic lesion. The cysticercus cyst was completely removed without complications (Figure 4). The patient presented a favorable evolution, recovering mobility of the left-sided hemiparesis and being discharged with treatment with albendazole.

**Figure 5:**
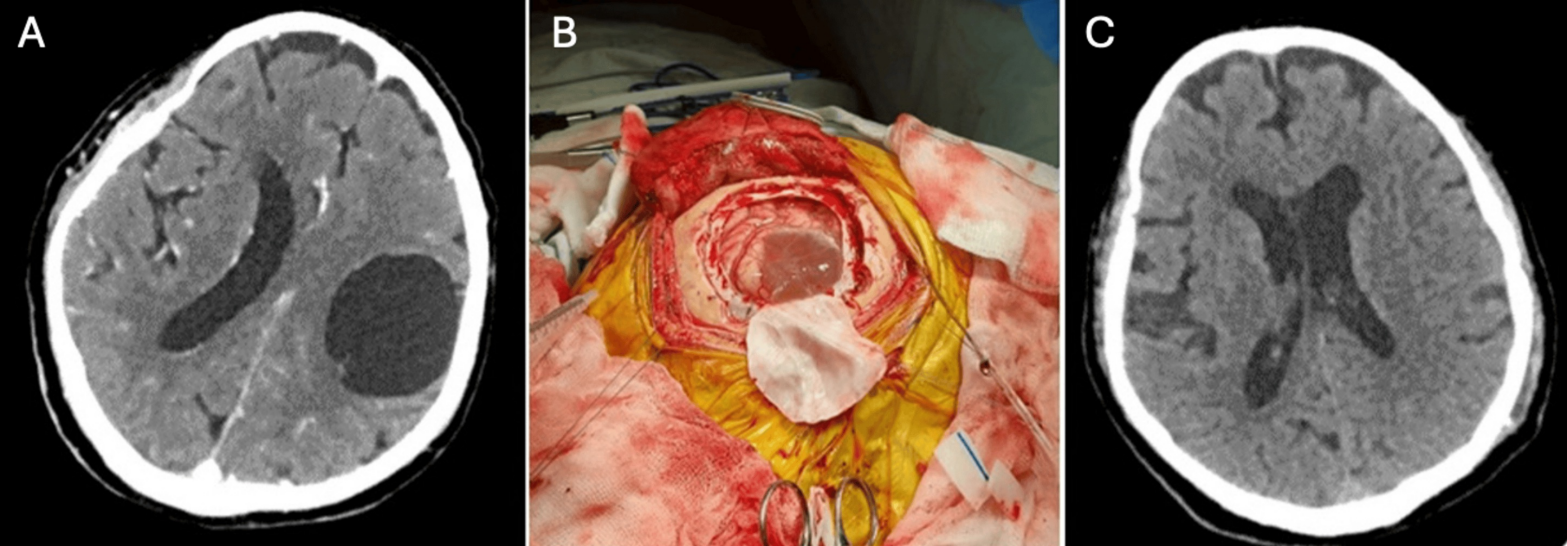
CT scan of the head shows a giant cysticercus cyst in the left frontoparietal region (A). Intraoperative image showing a cysticercosis cyst over the cerebral cortex (B). Control CT after surgery shows complete resection of the cysticercosis without complications (C) CT: computed tomography

The third case is a 76-year-old man from the southern coast of Peru who was admitted to the hospital with a disease duration of 10 years, characterized by sporadic headaches of moderate intensity and motor deficit characterized by weakness in the right upper limb, predominantly distal. A brain MRI was performed, showing calcified cysts of neurocysticercosis, receiving treatment with albendazole in two cycles, each lasting one month. The medical treatment did not improve his clinical condition, and a month before admission to the hospital, he presented with dysarthria, paralysis of the right upper limb, and severe weakness of the right lower limb that prevented him from standing. The patient finally entered the hospital due to generalized tonic-clonic seizures. A brain tomography was performed, which showed a 5 cm × 5 cm cystic brain lesion (Figure 5A). The Western blot study was positive for cysticercosis. The patient was scheduled for surgery, which involved a left frontal craniotomy and excision of the cystic lesion (Figure 5B). Postoperatively, the patient experienced favorable progress, with partial recovery of strength in the left side of his body. A new cycle of albendazole was prescribed upon discharge. In postoperative control, one month after surgery, the patient had no motor deficit. The control tomography showed complete removal of the cyst and the absence of complications (Figure 5C).

## Discussion

Neurocysticercosis is one of the major epidemiological problems in developing countries. This problem is worsening due to socioeconomic issues in these countries where healthcare systems are not suitable for the early diagnosis of these neurologic diseases [1,15].

Giant neurocysticercosis cysts are rare because smaller cysts can cause neurological symptoms like seizures or impairment of level of consciousness [1]. The progression of symptoms and the appearance of late motor deficits entail performing imaging studies, observing giant cystic lesions, and delaying the diagnosis [1,2]. This diagnosis delay is perceived in our three cases, in which the diagnosis was made after several weeks of symptoms starting.

Neurocysticercosis cysts are occasionally misdiagnosed as tumors because of the varying neuroimaging presentation. MRI imaging can demonstrate the various pathophysiological stages of neurocysticercosis, such as vesicular, colloidal, granular/nodular, and calcified stages [2]. In our three cases, giant cysts were observed in stage vesicular/colloidal. The serological diagnosis of neurocysticercosis is unsatisfactory due to high rates of false-positive and false-negative reactions. However, it may be helpful in the differential diagnosis of this disease, especially in endemic areas [3,16,17].

The purpose of surgical management of giant neurocysticercosis cysts is to reverse the associated neurological deficits and decrease elevated intracranial pressure [7,18]. This is achieved by total microsurgical excision of the cyst [19]. This technique must avoid damage to the surrounding brain tissue and exposure of the cystic fluid to the normal brain tissue.

In some cases, postoperative neurological deterioration has been attributed to the intraoperative rupture of the cyst due to the release of antigenic components that may cause severe inflammation throughout the CNS. Therefore, the use of the microscope and saline irrigation is suggested to avoid possible cyst rupture complications [20].

Antiparasitic drugs such as albendazole or praziquantel are the first-line therapy in most cases of neurocysticercosis. Even if the giant cyst is evacuated, these drugs should be administered to eradicate other possible systemic infections [1]. Our three reported cases have been surgically managed, obtaining good outcomes without major complications and a complete neurological recovery.

## Conclusions

Neurocysticercosis can present serious complications, so prevention of the disease, as well as early diagnosis, is essential. Although the treatment of choice is antiparasitic drugs, surgical removal may be indicated when intracranial hypertension and/or motor deficit is associated. As much as possible, we must avoid intraoperative rupture of the cyst and avoid damage the surrounding brain tissue irritation.
